# Accuracy of swept-source optical coherence tomography based biometry for intraocular lens power calculation: a retrospective cross–sectional study

**DOI:** 10.1186/s12886-019-1036-y

**Published:** 2019-01-24

**Authors:** Youngju An, Eun-Kyoung Kang, Hyojin Kim, Min-Ji Kang, Yong-Soo Byun, Choun-Ki Joo

**Affiliations:** 1 Catholic Institute for Visual Science, College of Medicine, The Catholic University of Korea, Seoul, Republic of Korea; 2 Thin Film Optics Laboratory, Department of Physics, Inha University, Incheon, Republic of Korea; 3 Department of Visual Optics, Division of Health Science, and Graduate School of Health and Welfare, Baekseok University, Cheonan, Republic of Korea; 4 Department of Ophthalmology and Visual Science, Seoul St. Mary’s Hospital, College of Medicine, The Catholic University of Korea, #505 Banpo-Dong, Seocho-Gu, Seoul, 137-040 Republic of Korea

**Keywords:** Intraocular lens power calculation, Cataract, Swept-source optical coherence tomography, Partial coherence interferometry

## Abstract

**Background:**

To evaluate the accuracy of biometric measurements by a swept-source optical coherence tomography (SS–OCT) based biometry for intraocular lens (IOL) power calculation.

**Methods:**

This retrospective observational study enrolled 431 patients undergoing cataract surgery. The charts were reviewed to investigate the failure rate of axial length (AL) measurement of the SS–OCT biometer, partial coherence interferometry (PCI), and A–scan ultrasonography (US) according to cataract type and severity. AL and keratometry in 164 eyes with the same IOL inserted were measured using the SS–OCT biometer, PCI, and A–scan US. The SRK/T formula was used to calculate IOL power. The mean absolute error (MAE) and percentage of eyes with a prediction error (PE) of ±0.50 D were compared.

**Results:**

The AL measurement failure rate was 0.00% for A–scan US, 2.32% for the SS–OCT biometer, and 15.31% for PCI. The number of eyes measured using three devices (SS–OCT biometer, PCI, and A–scan US) was 128 (Group A) and the number of eyes measured using two devices (SS–OCT biometer and A–scan US) was 36 (Group B). The score of posterior subcapsular opacity was significantly different between two groups (*p* < .001). The SS–OCT biometer and PCI showed significantly lower MAE compared to A–scan US in Group A (*p* = 0.027). Using SS-OCT biometer, MAE showed no significant difference between Group A (0.36 ± 0.27) and Group B (0.36 ± 0.31) (*p* = 0.785). Whereas, MAE of A-scan US was significantly higher than Group A (0.47 ± 0.39) in Group B (0.64 ± 0.36) (*p* = 0.023).

**Conclusions:**

Using biometry with advanced OCT is useful in clinical practice as it is more effective in obtaining biometric measurements in the eyes with PSC and provides accurate measurements for IOL power calculation regardless of cataract type and severity.

**Trial registration:**

Retrospectively registered. Registration number: KC16RISI1020. Registered 03 January 2018.

## Background

In the era of refractive cataract surgery, the achievement of a desired refractive outcome begins with intraocular lens (IOL) power calculation based on accurate biometry. The refractive status after cataract surgery is affected by various factors such as measurement error of the axial length (AL), measurement error of the corneal power, and the estimation of the pseudophakic anterior chamber depth (ACD) [1]. Of these, AL measurement accounts for 54% of the sources causing error in the refractive outcome in cataract surgery [1]. At present, the advent of partial interferometry (PCI) has reduced the effect of AL measurement errors by 36%, but ultrasonography (US) AL measurements still accounts for more than 50% of the total error in cataract surgery refraction results [2].

For many years, US had been the only technique used to measure AL in clinical practice [2]; however, recently, the use of optical biometry has increased due to advantages pertaining to non-invasiveness and inter-operator reproducibility. Introduced in 1999, IOLMaster (Carl Zeiss, Jena, Germany) is known to provide reliable measurements based on PCI with a 780-nm laser diode infrared light [3]. Introduced in 2009, the Lenstar LS 900 (Haag-Streit AG, Koeniz, Switzerland) is based on the principle of optical low-coherence reflectometry (OLCR) using 820-nm super-light emitting diodes. Lenstar LS 900 provides measurements of AL, keratometry (K), anterior chamber depth, and white-to-white, as well as central corneal thickness, pupil size, and lens thickness [4]. Nevertheless, the major limitation of these optical biometries is that measurement is impossible in patients with severe lens opacity. In previous studies, the AL measurement failure rates of PCI and OLCR in rural Chinese populations were reported as 37.84 and 35.47, respectively [5]. In particular, accurate measurement of AL in patients with posterior subcapsular cataract (PSC) remains a challenge [5].

The devices most recently introduced in clinical practice, e.g., Argos (Movu, Santa Clara, CA), IOLMaster 700 (Carl Zeiss Meditec, Jena, Germany), OA-2000 (Tomey, Nagoya, Japan), and others, are optical coherence tomography (OCT) systems based on swept source (SS) and are currently receiving attention because of their high-tissue penetration power. Argos involves biometry combined with SS OCT, is noninvasive, and provides increased confidence by generating real–time 2D images of the whole eye (from cornea to retina) during alignment [6]. Compared to conventional optical biometry, Argos has approximately a 30-times higher-speed swept-source laser centered at 1060 nm and an improved signal to noise ratio, providing a high AL-measurement success rate and high image quality [6].

Most previous studies using SS–OCT have evaluated repeatability and reproducibility [6–8], and studies on IOL power calculations, which is the main reason why they were developed, have received relatively less attention. In addition, few studies have evaluated the accuracy of IOL power calculations measured by the SS–OCT biometer in eyes with PSC. The objectives of the present study was to evaluate the accuracy of the measurements for IOL power calculation obtained with the SS–OCT biometer compared with conventional devices based on PCI feasibility.

## Patients and methods

In this retrospective cross–sectional study, the medical records of all patients diagnosed with cataract between August 2016 and November 2016 at Seoul St. Mary’s Hospital, Seoul, South Korea were reviewed. The study was approved by the Institutional Review Board of the Catholic University (No. KC16RISI1020). Patients with preoperative measurements using the SS–OCT biometer (Argos), PCI (IOLMaster, version 5.40), A–scan US (Axis nano, Quantel Medical, Clermont-Ferrand, France), and manual keratometry (OM-4; TOPCON Corp, Tokyo, Japan) were included in this study. One eye of each patient was included in the study. If both eyes met the inclusion criteria, one eye was selected randomly. Exclusion criteria included previous refractive surgery, keratoconus and any other corneal disease, impossible cooperation for testing due to retinal disease, or insufficient mental ability, and postoperative corrected distance visual acuity worse than 0.5 (Snellen 20/40). The research methods adhered to the tenets of the Declaration of Helsinki.

Cataract type and severity were graded according to the Lens Opacities Classification System III (LOCS ΙΙΙ) [9]. All patients underwent a slit–lamp evaluation conducted by a single experienced ophthalmologist, with the pupil dilated (Tropicamide 0.5% – phenylephrine 0.5% [Mydrin–P]). The types of cataracts were classified into four categories according to their opaque location; nuclear opalescence (NO), nuclear color (NC), cortical (C), and posterior subcapsular cataract (PSC). Also, the severity of cataracts was graded on a scale of 0.1 to 6.9 for NO and NC, and 0.1 to 5.9 for C and PSC, by comparison with a digital photograph of each lens with standard photographic transparencies. Further details can be found in previous literature [10].

All eyes were measured with the SS-OCT biometer, PCI, A–scan US, and manual keratometry on the same day without dilation. Measurements were taken by four experienced examiners, one for each instrument. All instruments were measured more than 3 times and the mean value was used. The SS-OCT biometer, PCI, and manual keratometry were used to fix the patient’s forehead and chinrests to each biometer, and the alignment was achieved with the patients fixed on a projected light at optical infinity. The A–scan US was performed at the end of the procedure because it may affect other tests with corneal contact. After the topical anesthetic (Alcaine 0.5%) was installed, the 11-MHz probe was contacted with the cornea to measure AL.

Phacoemulsification was performed by the same surgeon (C.–K.J.) through a 2.2-mm self-sealing temporal clear corneal incision under topical anesthesia (topical lidocaine 4% and proparacaine hydrochloride 0.5%). Diverse IOL models were inserted in the bag depending on patient condition and need (e.g., Toric and multifocal IOLs). Because constant optimization should be performed separately for each IOL model [11], only the IOL model used in the largest sample of patients was selected for subset analysis.

The charts were reviewed to calculate AL measurement failure rates for SS–OCT biometer, PCI, and A–scan US. Of the patients with the same IOL inserted, if PCI was measured (Group A), AL and K measured using SS–OCT biometer, PCI, and A–scan US were collected; if PCI was not measured (Group B), AL and K measured using the SS–OCT biometer and A–scan US were collected. Because A–scan US does not have a function to measure K, manual keratometry values were used to calculate intraocular lens power.

IOL power calculation was performed based on the SRK/T formula. The optimized lens constants used in the study were 118.02, 118.04, and 118.0 for SS–OCT biometrics, IOLMaster, and A–scan US, respectively. A final evaluation was performed by collecting the results of the subjective spherical equivalent refractive outcome at 2 months postoperatively. The mean prediction error (PE) was obtained by subtracting the predicted spherical equivalent refraction from the postoperative subjective spherical equivalent refraction. Thus, a positive refractive PE reflected hyperopic refractive outcome. The mean absolute error (MAE), the median absolute error, and the percentage of eyes with PE within ±0.50 D were calculated.

### Statistical analysis

Statistical analysis was performed using SPSS version 18.0 software (IBM Corp., Armonk, NY). All data were expressed as mean ± standard deviation. Normality was verified using the Kolmogorov-Smirnov test. Mann**–**Whitney U test was used to compare two independent groups, and Wilcoxon signed rank test was used for pairwise comparisons. The Kruskal-Wallis method was used for nonparametric data among three groups. All tests were 2-tailed, and a *p* value < .05 was considered to be statistically significant.

## Results

Four hundred thirty–one eyes of 431 patients diagnosed with cataract during the recruitment period were included in the study. The mean age was 66.70 ± 10.54 (SD) years (range 23 to 87 years), and 281 eyes (65.20%) belonged to women. The IOL model used in the largest patient sample was Precizon Monofocal 560 (Ophtec, Inc., Boca Raton, FL). Of the 431 consecutive patients, it was possible to evaluate at 2 months postoperatively the subjective spherical equivalent refractive outcome after the insertion of the Precizon Monofocal 560 in 164 eyes. Of these, 128 eyes (Group A) were able to measure axial length using the A–scan US, the SS-OCT biometer, and PCI. Because 36 eyes (Group B) could not be measured using PCI, axial length was measured using A-scan US and SS–OCT biometer.

### Measurement failure rate of axial length

A total of 431 eyes were used to calculate the AL measurement failure rate for each device. All of these eyes could be measured with A–scan US. Of the 426 eyes for which LOCS ΙΙΙ grading was possible, both SS–OCT biometry and PCI measured in 365 eyes, SS–OCT biometry measured but PCI not measured in 56 eyes, and both SS–OCT biometry and PCI were not measured in 5 eyes. In another 5 eyes, LOCS ΙΙΙ grading was impossible due to mature cataract, in which cases measurement was not performed either with the SS–OCT biometer or with PCI. Thus, the overall measurement failure rate was 0.00% (0 eyes) for A–scan US, 2.32% (10 eyes) for the SS–OCT biometer, and 15.31% (66 eyes) for PCI.

### Lens opacity

The cataract type and severity were graded according to LOCS ΙΙΙ in 164 eyes. The mean ± standard deviation (median) scores of NO, NC, C and PSC were 2.83 ± 0.64 (3.00), 2.80 ± 0.66 (3.00), 2.08 ± 1.41 (3.00) and 0.52 ± 1.03 (0.00) for Group A and 2.69 ± 1.09 (2.50), 2.64 ± 1.13 (2.00), 1.89 ± 0.90 (2.00) and 3.72 ± 1.06 (4.00) for Group B, respectively. The scores of NO, NC, and C were not statistically different between the two groups, but PSC score was statistically significant (*p* < .001; Table 1).

**Table 1:** Comparison of lens opacity

LOCS ΙΙΙ	Group A (n = 128)	Group B (n = 36)	*p*–value^*^
Nuclear opalescence	2.83 ± 0.64 (3.00)	2.69 ± 1.09 (2.50)	0.122
Nuclear color	2.80 ± 0.66 (3.00)	2.64 ± 1.13 (2.00)	0.103
Cortical	2.08 ± 1.41 (3.00)	1.89 ± 0.90 (2.00)	0.158
Posterior subcapsular	0.52 ± 1.03 (0.00)	3.72 ± 1.06 (4.00)	*p* < .001

Values are presented as mean ± standard deviation (median)

Group A; PCI feasible, Group B; PCI unfeasible

LOCS ΙΙΙ = Lens Opacities Classification System ΙΙΙ

^*^p–value is for Mann**–**Whitney U test

### Biometry

Table 2 shows the comparison of biometric measurements between devices according to PCI feasibility. In Group A, AL and K did not show any significant difference between the three devices. In Group B, the SS–OCT biometer and A–scan US showed a significant difference in AL (*p* < .001), while the SS–OCT biometer and Manual K did not show a significant difference in K.

**Table 2:** Comparison of corneal power and axial length

	SS–OCT biometer	PCI	A–scan US	*p*–value
Group A (n = 128)				
Corneal power (D)	44.14 ± 1.47	44.16 ± 1.50	44.04 ± 1.50^‡^	0.811^*^
Axial length (mm)	24.56 ± 2.16	24.58 ± 2.22	24.49 ± 2.19	0.935^*^
Group B (n = 36)				
Corneal power (D)	44.01 ± 1.92	N/A	44.03 ± 1.88^‡^	0.924^†^
Axial length (mm)	25.18 ± 2.86	N/A	25.02 ± 2.82	*P* < .001^†^

Values are presented as mean ± standard deviation

Group A; PCI feasible, Group B; PCI unfeasible

*D* diopters, *SS–OCT* swept–source optical coherence tomography, *PCI* partial coherence interferometry, *US* ultrasonography

^*^p–value is for Kruskal**–**Wallis test

^†^*p*–value is for Wilcoxon signed rank test

^††^Measurement using manual keratometry

### Refractive error

Table 3 shows the results of evaluating the accuracy of the refractive error prediction. In Group A, the SS–OCT biometer and PCI showed statistically significantly lower MAE than A–scan US (*p* < .05), and there was no significant difference between the SS–OCT biometer and PCI. In Group B, the SS–OCT biometer showed a statistically significantly lower MAE than A–scan US (*p* < .001).

**Table 3:** Comparison of refractive outcomes of intraocular lens power calculation using the three devices in SRK/T formula

	Group A (n = 128)			Group B (n = 36)	
	SS–OCT biometer	PCI	A–scan US	SS–OCT biometer	A–scan US
Optimized constant	118.02	118.04	117.90	118.02	117.90
PE (D)	0.00 ± 0.44	0.00 ± 0.46	−0.02 ± 0.61	0.00 ± 0.47	−0.01 ± 0.66
MAE (D)	0.36 ± 0.27^†^	0.39 ± 0.30^†^	0.47 ± 0.39	0.36 ± 0.31	0.64 ± 0.36
*p***–**value	0.027^*^			*p* < .001^‡^	
MedAE (D)	0.31	0.32	0.40	0.32	0.60
Eye within (%)					
≤ ±0.25 D	41.44	42.97	36.72	47.22	22.22
≤ ±0.50 D	71.09	67.19	60.16	72.22	47.22
≤ ±1.00 D	95.31	95.31	90.63	94.44	83.33

Values are presented as mean ± standard deviation

Group A; PCI feasible, Group B; PCI unfeasible

*D* diopters, *PE* prediction error, *MAE* mean absolute error, MedAE median absolute error, *SS-OCT* swept-source optical coherence tomography, *PCI* partial coherence interferometry, *US* ultrasonography

^*^p**–**value is for Kruskal**–**Wallis test

^†^Same letters indicate no statistical significance based on Bonferroni’s method

^††^*p***–**value is for Wilcoxon signed rank test

**Fig. 1:**
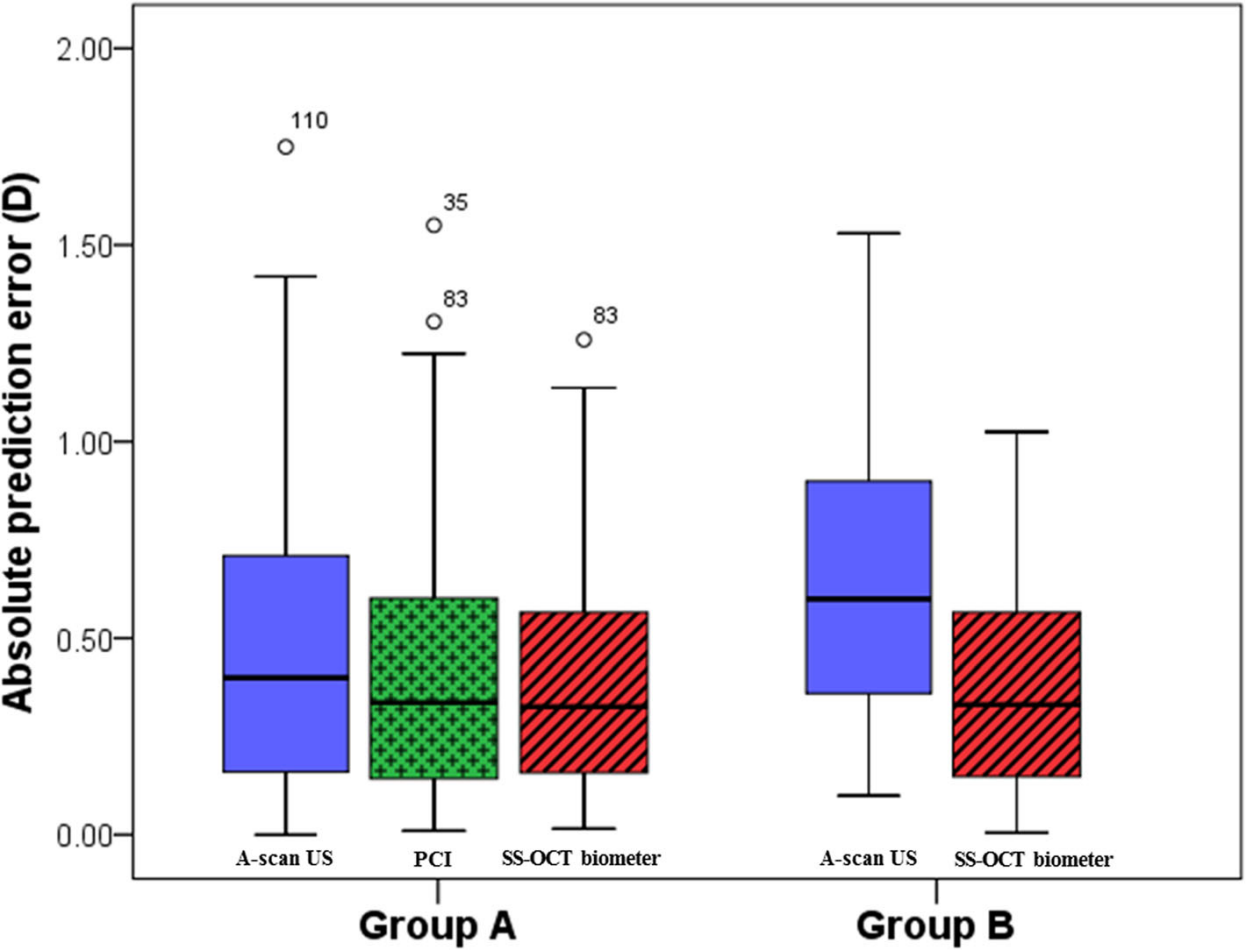
The distribution of absolute prediction error using A-scan US, PCI, and SS–OCT biometer in SRK/T formulas. Group A; PCI feasible, Group B; PCI unfeasible (US = Ultrasonography, PCI = Partial coherence interferometry, SS–OCT = swept-source optical coherence tomography, NS = not significant) ^*^p–value is for Mann–Whitney U test.

The MAE of the SS-OCT biometer was not significantly different between Group A (0.36 ± 0.27) and Group B (0.36 ± 0.31) (*p* = 0.736; Fig. 1), and the percentage of eyes with a PE of ±0.50 D or less was 71.09 and 72.22% in Group A and Group B, respectively. On the other hand, the MAE of A-scan US was significantly different between Group A (0.47 ± 0.39) and Group B (0.64 ± 0.36) (*p* = 0.007; Fig. 1), and the percentage of eyes with a PE of ±0.50 D or less was 60.16 and 47.22% in Group A and Group B, respectively.

## Discussion

Accurate biometric measurements are critical for IOL power calculations. Currently, optical biometry is considered to be the most accurate method among biometers, and the majority of optical biometries scan the eye using PCI or time-domain OCT technology [12]. However, the major limitation of these technologies is that light can only penetrate media up to a specific opacity [12]. In the present study, the SS–OCT biometer showed a high acquisition rate of 97.68%, and the percentage of eyes with a PE of ±0.50 D or less was 71.09 and 72.22% in Group A and Group B, respectively. This is above the 55% value established as the benchmark standard by the National Health Service of the United Kingdom [13].

Various AL measurement failure rates have been reported so far due to methodological differences in clinical settings (e.g., patient’s cataract type and severity, approach and version of the measurement device, and others). The AL measurement failure rates of IOLMaster have been reported to be 10–20% in previous studies [14–17], but these studies did not classify cases according to grade of cataract density and morphology. A study which proposed cutoff values of cataract morphology and severity to measure AL has shown that IOLMaster v5.4 could not be used for measurements in cases of mature cataract and a LOCS ΙΙΙ P-scale value of 3.5 or higher [18]. The reason for such high AL measurement failure rate in PSC is that the location of opacity in the posterior pole is adjacent to the lens’ nodal point and, accordingly, it affects more the passing of light rays compared to other types of morphology [5]. In clinical practice, PSC is known to cause visual disability regardless of the extent of opacity [19].

In this study, the AL measurement failure rate of Argos based on SS–OCT was 2.32%, which was similar to that reported in previous studies [6]. In addition, Argos can be measured at an N-scale value of 6 and P-scale value of 5. SS–OCT is the most recently introduced OCT technology that uses a fast cycle and tunable wavelength laser source to sequentially scan the anterior segment or even the whole eye [12]. In particular, Argos uses a 1060-nm wavelength and 20-nm bandwidth swept light source technology to collect two-dimensional OCT data of the whole eye [6]. The use of two-dimensional OCT data improves the repeatability of the measurement as well as the success rate in AL measurement [6].

In this study, AL was measured in the order of A–scan US, SS–OCT biometer, and PCI, but there was no statistical significance. It is known that US is reflected from the internal limiting membrane, while light in laser interferometry is reflected from the retinal pigment epithelium [20], which may result in difference corresponding to the retinal thickness in the fovea (130 μm). For PCI, the group refractive index (1.3549) is used to calculate the AL, while the SS–OCT biometer uses different refractive indices depending on the ocular media (1.376 for the cornea, 1.336 for the aqueous and vitreous, and 1.410 for the lens) [6]. Therefore, we expected that there would be a significant difference in AL measurement between devices, but the results showed no significant difference. This result can be attributed to the fact that numerous individuals with normal axial length were included in this study.

Although there was no significant difference in the biometric measurements among three devices, the SS–OCT biometer and PCI showed a lower MAE and a higher PE of ±0.50 D or less than the A–scan US. These results can be considered as a cause of the difference in the agreement of AL measurements. In Group B with relatively high PSC score, the SS–OCT biometer and A–scan US AL measurements were significantly different and the inter-device agreement was low. The SS–OCT biometer showed comparable MAEs in Group A (0.36 ± 0.27) and Group B (0.36 ± 0.31), while the A-scan US showed a greater MAE in Group B (0.64 ± 0.36) than Group A (0.47 ± 0.39). These results suggest high accuracy of SS–OCT technology in PSC as well as normal density cataracts. In a lens with dense opacity, the average US velocity decreases and can cause an AL measurement error [21]. Also, Ueda et al. [21] reported that the IOLMaster’s measurements were affected by cataract density but that the US was more affected in terms of accuracy. It is because in US the examiner estimates the location of the optical axis in the eye and performs a manual alignment, unlike optical biometry in which a more accurate alignment is guaranteed by using a visual axis while the patient stares at a light spot [21].

The main limitations of this study are as follows. First, we did not investigate other formulas (e.g., Haigis, Olsen, and Barrett Universal) using the SS–OCT biometer, although they provide additional measurements such as ACD or lens thickness. However, the comparison of formulas was not the purpose of this study. Second, although the immersion-type US is known to have better reproducibility, we used the applanation type because of the characteristics of the retrospective study. However, the A–scan US measurement was performed by an examiner with an experience of over 3 years, and AL measurements by applanation US obtained by experienced operators are reported to have less difference and lower variability in the difference compared to AL measurements obtained with IOLMaster [22].

## Conclusions

PCI has been regarded as an excellent noncontact measurement method in the eyes of cataract patients. Nevertheless, US biometry is still required for cases with PSC [23]. SS–OCT biometer and PCI There was no statistically significant difference in MAE between the SS–OCT biometer and PCI, but the measurement failure rate was lower for the SS–OCT biometer (2.32%) than for PCI (15.31%). Moreover, the SS-OCT biometer maintained the accuracy of the measurements for IOL power calculation without statistically significant difference from Group A in Group B. Whereas, A-scan US showed higher error than Group A in Group B. Thus, Using biometry with advanced OCT is useful in clinical practice as it is more effective in obtaining biometric measurements in the eyes with PSC and predictable refraction results compared to conventional devices.

## Abbreviations

ACD: Anterior chamber depth
AL: Axial length
C: Cortical
IOL: Intraocular lens
K: Keratometry
LoA: Limits of agreement
LOCS ΙΙΙ: Lens Opacities Classification System III
MAE: Mean absolute error
NC: Nuclear color
NO: Nuclear opalescence
OCT: Optical coherence tomography
OLCR: Optical low-coherence reflectometry
PCI: Partial coherence interferometry
PE: Prediction error
PSC: Posterior subcapsular cataract
SS: Swept source
US: Ultrasonography
